# Removal of Pharmaceutical Micropollutants with Integrated Biochar and Marine Microalgae

**DOI:** 10.3390/microorganisms9010004

**Published:** 2020-12-22

**Authors:** Amin Mojiri, Maedeh Baharlooeian, Reza Andasht Kazeroon, Hossein Farraji, Ziyang Lou

**Affiliations:** 1 Department of Civil and Environmental Engineering, Graduate School of Advance Science and Engineering, Hiroshima University, Higashihiroshima 739-8527, Japan; 2 Department of Marine Biology, Faculty of Marine Science and Oceanography, Khorramshahr University of Marine Science and Technology, Khorramshahr 669, Iran; bbenicka@yahoo.com; 3 Faculty of Civil Engineering, Xi’an University of Architecture and Technology, Xi’an 710055, China; reza.andasht@gmail.com; 4 School of Physical and Chemical Sciences, University of Canterbury, Christchurch 8140, New Zealand; faraji6211@gmail.com; 5 School of Environmental Science and Engineering, Shanghai Jiao Tong University, Shanghai 200240, China; louworld12@sjtu.edu.cn

**Keywords:** biochar, carbamazepine, emerging micropollutant, microalgae, sulfamethazine, tramadol

## Abstract

Using microalgae to remove pharmaceuticals and personal care products (PPCPs) micropollutants (MPs) have attracted considerable interest. However, high concentrations of persistent PPCPs can reduce the performance of microalgae in remediating PPCPs. Three persistent PPCPs, namely, carbamazepine (CBZ), sulfamethazine (SMT) and tramadol (TRA), were treated with a combination of *Chaetoceros muelleri* and biochar in a photobioreactor during this study. Two reactors were run. The first reactor comprised *Chaetoceros muelleri*, as the control, and the second reactor comprised *Chaetoceros muelleri* and biochar. The second reactor showed a better performance in removing PPCPs. Through the response surface methodology, 68.9% (0.330 mg L^−1^) of CBZ, 64.8% (0.311 mg L^−1^) of SMT and 69.3% (0.332 mg L^−1^) of TRA were removed at the initial concentrations of MPs (0.48 mg L^−1^) and contact time of 8.1 days. An artificial neural network was used in optimising elimination efficiency for each MP. The rational mean squared errors and high *R*^2^ values showed that the removal of PPCPs was optimised. Moreover, the effects of PPCPs concentration (0–100 mg L^−1^) on *Chaetoceros muelleri* were studied. Low PPCP concentrations (<40 mg L^−1^) increased the amounts of chlorophyll and proteins in the microalgae. However, cell viability, chlorophyll and protein contents dramatically decreased with increasing PPCPs concentrations (>40 mg L^−1^).

## 1. Introduction

Industrial and agricultural activities are the main sources of water pollution around the world [1]. In the scientific literature and CAS registry, more than 150 million inorganic and organic pollutants have been recorded [2]. The continuous input of microcontaminants to water bodies is a growing environmental problem given that many of these microcontaminants are non-biodegradable, persistent and bioaccumulative [3]. Among the important categories of these pollutants are emerging micropollutants, which can be detected in the environment at trace concentrations. These contaminants comprise personal care products, pharmaceuticals, pesticides, metallic trace elements and industrial chemicals [4]. Vakili et al. [5] demonstrated that conventional treatment techniques used by municipal wastewater treatment plants have failed to eliminate emerging micropollutants completely.

Pharmaceuticals and personal care products (PPCPs) constitute an important class of emerging micropollutants. Tons of PPCPs are annually produced, consumed and finally discharged into the environment [6]. Therefore, some of the most persistent PPCPs, namely, carbamazepine (CBZ), sulfamethazine (SMT) and tramadol (TRA), were investigated in the present study. Yentür and Dükkancı [7] stated that CBZ, as an antiepileptic drug, has been usually used to treat epilepsy and bipolar disorder. Approximately 10% of CBZ may be eliminated from wastewater through conventional treatment [7]. SMT is a common sulfonamide antibiotic and used in animal husbandry and aquatic farming. Approximately 50% of consumed SMT remains unmetabolised in parent animals and may be excreted to water bodies [8]. Another investigated PPCP in this study is TRA, which is a painkiller and opioid analgesic [9]. It is not completely eliminated by wastewater treatment plants and usually discovered in effluents after treatment and in surface waters [10]. Consequently, the removal of these micropollutants has become a worldwide concern [3]. Thus, several physicochemical and biological methods for removing PPCPs from water bodies have been reported [11]. One of several techniques for treating microcontaminants is bioremediation, a low-cost and environmentally friendly method. It is a procedure that involves the use of microorganisms, such as bacteria, fungi and algae, in degrading and transforming contaminants into less toxic forms [12]. Microalgae have attracted the attention of researchers in the field of PPCP removal through bioremediation [13]; microalga systems have a dual capability to treat wastewater efficiently and produce biomass for the production of biofuel, biofertiliser or other useful products [14].

Robledo-Padilla et al. [15] expressed that several marine or freshwater microalgae can be used for the removal of organic pollutants. Jiménez-Bambague et al. [16] removed 30–70% of PPCPs from domestic wastewater under tropical conditions by using green microalgae. Meanwhile, the capability of the marine diatom *Chaetoceros muelleri* to remove PPCPs has not been thoroughly explored in previous studies. Minggat et al. [17] stated that *Chaetoceros muelleri* is frequently used in aquacultural feed and is well known for its fast growth and easy maintenance. Wang et al. [18] stated that *Chaetoceros muelleri* is one of the suitable microalgae for large-scale biomass and lipid production. Karthikeyan et al. [19] used *Chaetoceros* sp. in macronutrient removal from wastewater. Mulla et al. [20] showed that hydraulic retention time, high concentration of PPCPs and seasonality could affect the efficiency of systems in removing several microcontaminants. To reduce the effects of these factors on microalgal activities, we added biochar to our system.

Biochar is a carbonaceous material produced from biomass feedstock through thermochemical decomposition in the presence of little oxygen or in the absence of oxygen [21]. Biochar is a promising adsorbent for low-cost wastewater treatment. It can be integrated into different treatment techniques to enhance the performance of a system [22]. Gorovtsov et al. [23] demonstrated that biochar could enhance the organism growth and activities by supplying nutrients and immobilising organisms on its surface.

Organic microcontaminants in aquatic environments can cause toxic effects on microorganisms, such as microalgae [24]. Therefore, most researchers [25,26,27] have attempted to evaluate the individual effects of organic microcontaminants on microalgae. Meanwhile, the combined effects of some emerging micropollutants on marine diatoms have not been extensively explored.

Therefore, the objectives of the current study were as follows: Firstly, an integrated system comprising biochar and *Chaetoceros muelleri* (marine diatom) was designed as a photobioreactor to remove PPCPs from synthetic wastewater, and the performance of the system was optimised using the response surface methodology (RSM) and an artificial neural network (ANN). The currently reported system has not been described in previous researches. Secondly, the effect of PPCPs on *Chaetoceros muelleri* was investigated.

## 2. Materials and Methods

CBZ, SMT and TRA of ≥98% purity (Table 1), distilled water and methanol were supplied by Sigma-Aldrich Co (Petaling Jaya, Malaysia). Stock solutions (1 g L^−1^) were prepared by individually dissolving the compounds in distilled water [28]. *Chaetoceros muelleri* was obtained from the photobioreactor in our laboratory.

### 2.1. Experimental Setup

**Figure 1:**
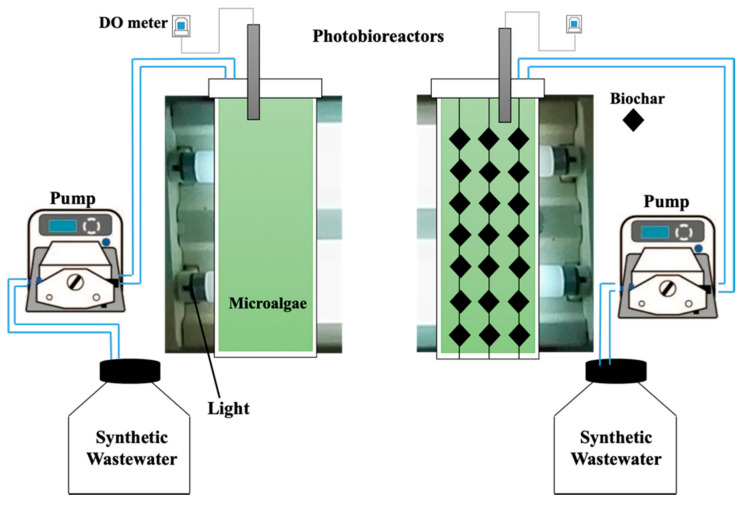
Schematics of the photobioreactors.

*Chaetoceros muelleri* was cultivated (Supplementary Materials, Figure S1) according to the method described by González-González et al. [30]. Briefly, the algae were cultivated in a F/2 medium in artificial seawater under constant light of around 66 µmol photons m^−2^ s^−1^ [31] at 25 °C. Then, the microalgae were transferred to an eight L bubble column photobioreactor under white fluorescent light illumination (66 µmol photons m^−2^ s^−1^) at room temperature. Two photobioreactors (Figure 1) were used. One of the photobioreactors contained *Chaetoceros muelleri* (first reactor), and another contained *Chaetoceros muelleri* and 20 g L^−1^ of biochar (biochar dose was selected according to preliminary experiments and hanged in the reactor; second reactor). On the basis of preliminary experiments, aeration rate was set at 0.4 L/min [32] in both reactors. Synthetic aqueous solution was produced by dissolving PPCPs and artificial seawater. The concentrations of PPCPs ranged from 0.2 mg L^−1^ to 1 mg L^−1^. According to preliminary experiments, hydraulic retention time was set at 2.8 days. This setup is in line with the findings of Jiménez-Bambague et al. [16].

### 2.2. Organic Micropollutant Measurements and Biochar Characteristic Monitoring

A high-pressure liquid chromatograph (LC-20AT, Shimadzu International Trading Co., Ltd., Tokyo, Japan) with a UV detector was used in monitoring the concentrations of the organic micropollutants. The mobile phases were acetonitrile and NaH_2_PO_4_ in a ratio of 40/60. The limit of detection was determined using the expression 3σ/s, where *σ* is the standard deviation of the peak and *s* defines the slope of the corresponding calibration curve [28]. Biochar (derived from agricultural wastes) was used in this study. BET surface analysis was conducted using autosorb (Quantachrome AS1WinTM-automated gas-sorption apparatus, Boynton Beach, FL, USA).

### 2.3. Optimisation Analysis

PPCP removal efficiency was assessed using the following equation (Equation (1)).

(1) $$\mathrm{Removal}\;\left(\%\right)=\;\frac{Initial\;concentration\;\left(mg\;L^{-1}\right)-Final\;concentration\;\left(mg\;L^{-1}\right)}{Initial\;concentration\;\left(mg\;L^{-1}\right)}\times 100$$

RSM and central composite design were used in optimising the reactors’ efficiency in eliminating PPCPs, reaction (contact) time (0.5–9 days) and the initial concentrations of PPCPs (0.2–1 mg L^−1^). In this step, the Design Expert Software (Version 10) was used. Each factor had three levels, and thus a quadratic model was considered a suitable model (Equation (2)) [33].

(2) $$Y=\;\beta_{0}+\overset{k}{\underset{j=1}{\sum }}\beta_{j}X_{j}\;\overset{k}{\underset{j=1}{\sum }}\beta_{jj}X_{j}^{2}\underset{j=1}{\sum }\overset{k}{\underset{j>1}{\sum }}\beta_{jj}X_{j}X_{j}+e$$

where, *Y* is response, *ß*_0,_ and *ß_i_* are fixed coefficients, *ß_j_* and *ß_ij_* interface coefficients, *X_i_* and *X_j_* are variables and *e* represents error.

Furthermore, ANN was used in optimising PPCP removal in each photobioreactor. Optimisation was implemented in MATLAB R2015a. The topology of the ANN, containing the input, hidden and output layers, is shown in Figure S2. In this study, the initial concentrations of PPCPs and reaction time were two neurons of input. The five neurons in the hidden layer and one neuron in the output layer were considered in the ANN. *R*^2^ and mean squared errors (*MSE*; Equations (3) and (4)) were evaluated for the monitoring of model performance [34]. Approximately, 60%, 20% and 20% of data were considered in training, validation and testing, respectively.

(3) $$R^{2}=1\;-\;\frac{\sum_{i=1}^{N}\left(|{ỿ}_{prd,i}\;-\;{ỿ}_{exp,i\;}|\right)}{\sum_{i=1}^{N}(|{ỿ}_{prd,i}-{ỿ}_{m}|)}$$

(4) $$MSE=\;\frac{1}{N}\sum_{i=1}^{N}{\left(|{ỿ}_{prd,i}-{ỿ}_{exp,i}|\right)}^{2}$$

where by *y_prd,i_* is the anticipated value of the ANN model, *y_exp,i_* denotes the experimental value and *N* and *y_m_* denotes the number of data and the average of the experimental values, respectively.

### 2.4. Effects of Pharmaceuticals and Personal Care Products on Microalgae

To monitor the effects of pharmaceutical micropollutants on microalgae, we carried out the batch experiments according to the methods described by Tsiaka et al. [27]. Briefly, after the cultivation of the microalgae in the F/2 medium, a suitable amount of biomass (1 × 10^4^ cells mL^−1^) was transferred to a conical sterilised flask series and then exposed to various concentrations of TRA, SMT and CBZ (0–100 mg L^−1^) for 24–96 h under constant light of 66 µmol photons m^−2^ s^−1^. Total protein was measured at the wavelength of 595 nm with a UV-Vis spectrophotometer according to the methods described by Chia et al. [35] Carotenoids were analysed with a spectrophotometer (UV-1601PC, Shimadzu, Tokyo, Japan) at 470, 630 and 664 nm according to the methods described by Wang et al. [18]. Homogenous suspensions of the samples at 100 mL each were centrifuged at 6000× *g* for 12 min, and the obtained cell pellets were resuspended in 20 mL of acetone. The solvent biomass mixture was incubated at 45 ± 50 °C in a water bath for 2 h with shaking. The following equations (Equations (5)–(7)) [18] were used in calculating carotenoid content.

Chlorophyll *a* (µg mL^−1^) = (11.47A_664_) − (0.4A_630_) (5)

Chlorophyll *c* (µg mL^−1^) = (24.36A_630_) − (3.73A_664_) (6)

C_X+c_ = (1000A_470_ − 2.27C_a_ − 81.4C_b_) (7)

where, absorbance levels at 470, 664 and 630 nm are indicated by A_470_, A_664_ and A_630_, respectively.

Optical cell density of 680 nm (OD_680_) was used in monitoring cell growth [36], and then cytotoxicity assay was calculated using Equation (8) [37].

(8) $$\mathrm{Cell}\;\mathrm{viability}\;\left(\%\right)=\;\frac{A_{E}-A_{B}}{A_{C}-A_{B}}\;\times 100$$

where, the mean of absorbance levels of the blank and control cells are indicated by *A_B_* and *A_C_*, respectively, and *A_E_* shows the mean of absorbance of the cells exposed to the PPCPs.

### 2.5. Adsorption Isotherm for Micropollutant Removal by Biochar

As batch experiments, adsorption isotherm study was performed in beakers containing PPCPs (0.5 mg L^−1^) and different doses of biochar (0–25 g L^−1^). The beakers were shaken at 200 rpm for 24 h. Then, Equation (9) was used in evaluating adsorption capacity (q_e_, mg g^−1^).

(9) $$q_{e}=\;\frac{\left(C_{O}-C_{eq}\right)V}{M_{s}}$$

where, *V* denotes volume (L), *M_s_* denotes adsorbent mass (g) and *C*_0_ and *C_e_* are the initial and final concentrations of PPCPs, respectively.

## 3. Results and Discussion

**Figure 2:**
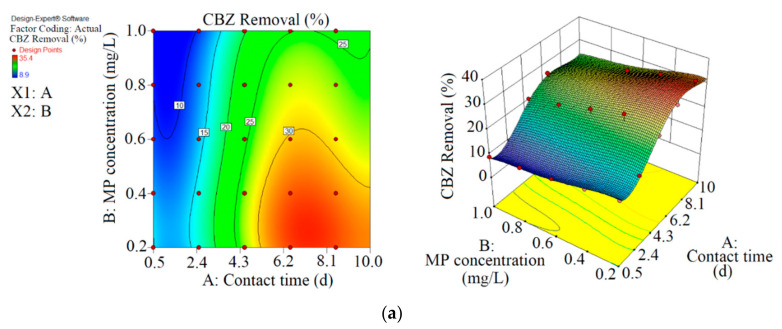

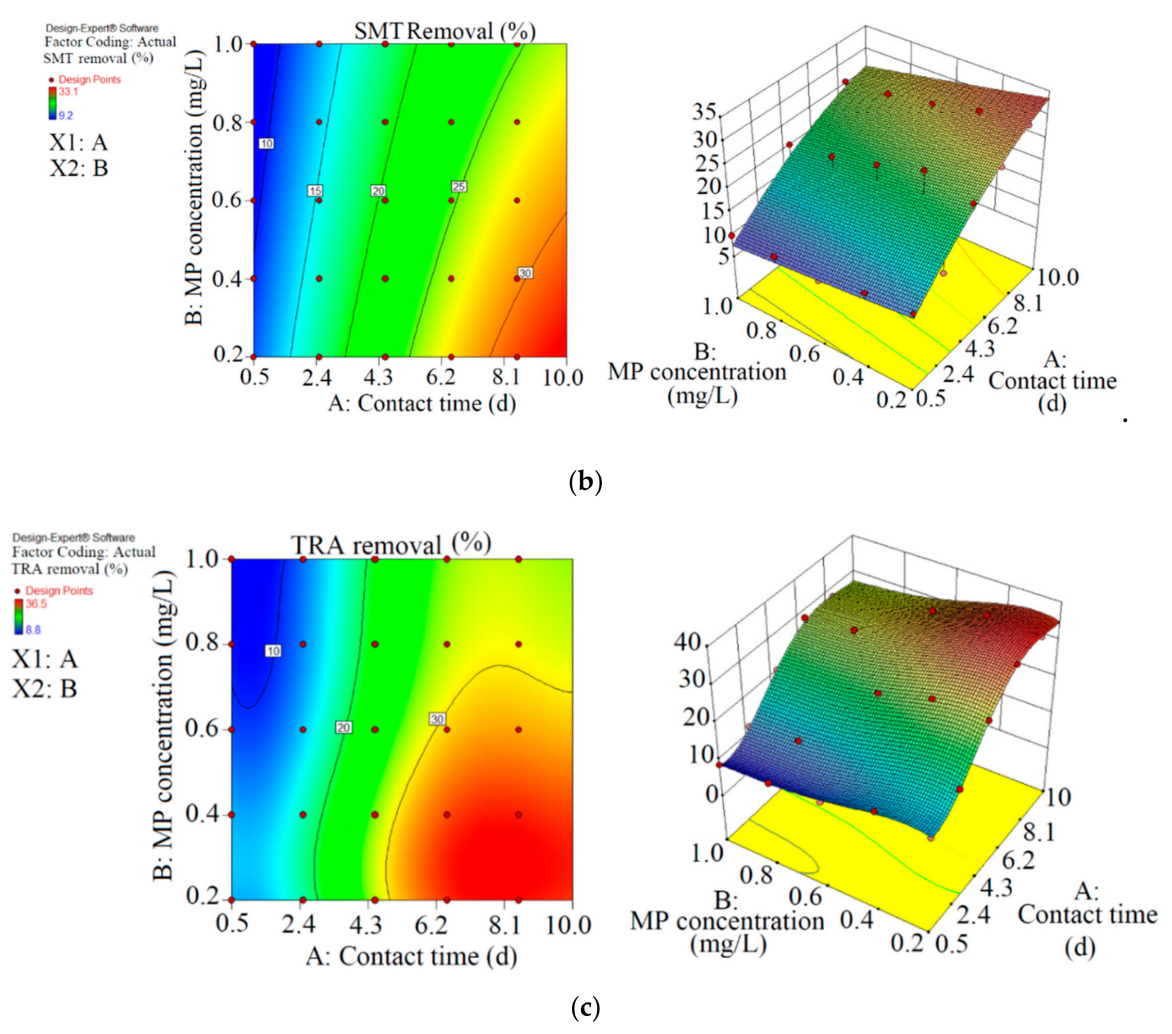
3D plots for the removal of CBZ (**a**), SMT (**b**) and TRA (**c**) by the first reactor based on the RSM.

**Figure 3:**
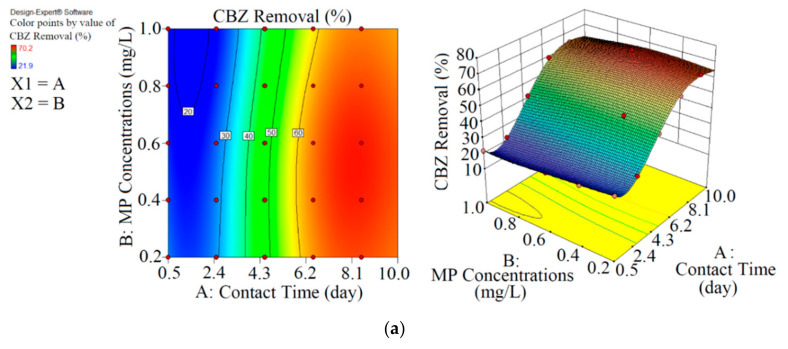

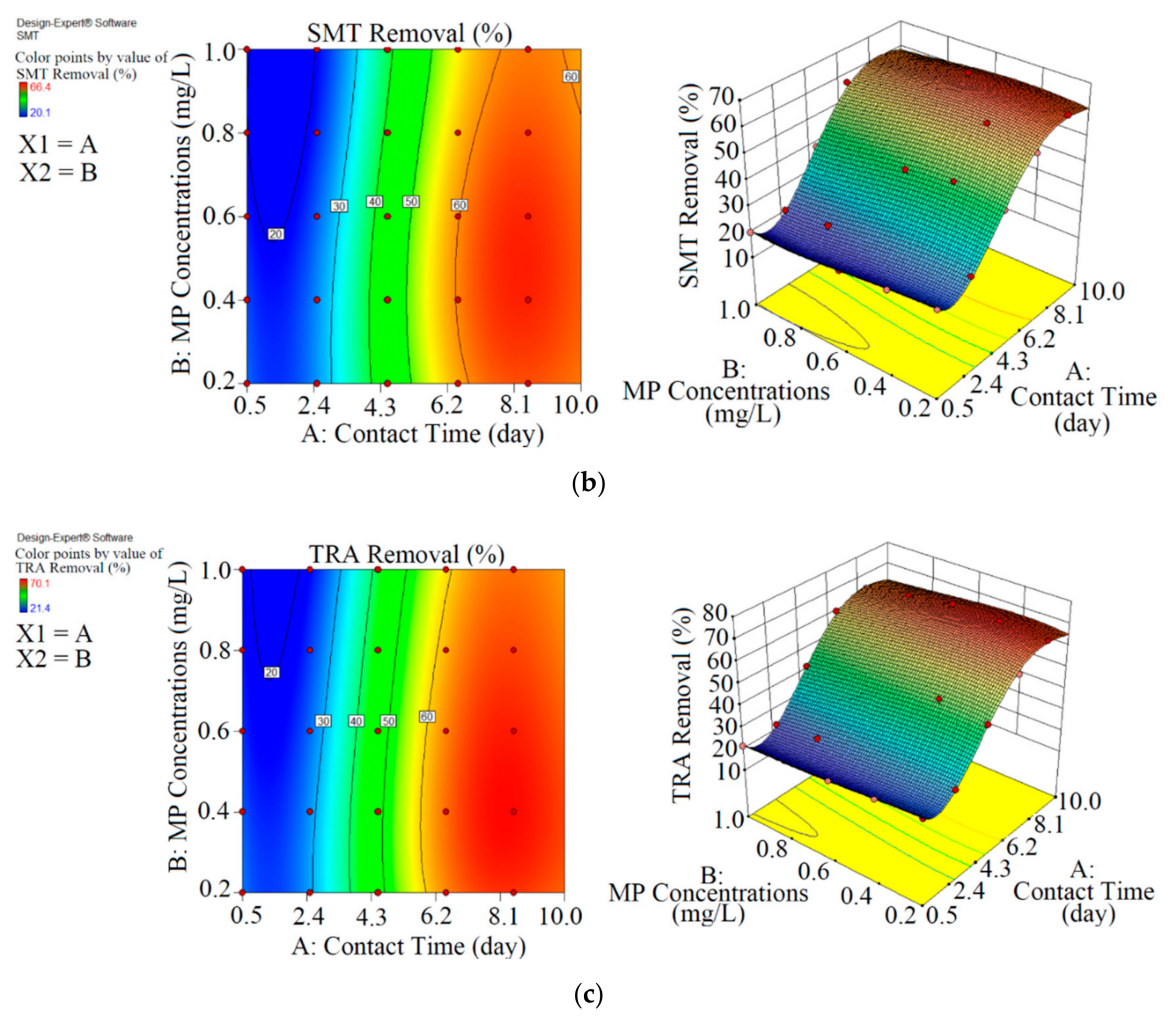
3D plots for the removal of CBZ (**a**), SMT (**b**) and TRA (**c**) in the second reactor based on the RSM.

**Figure 4:**
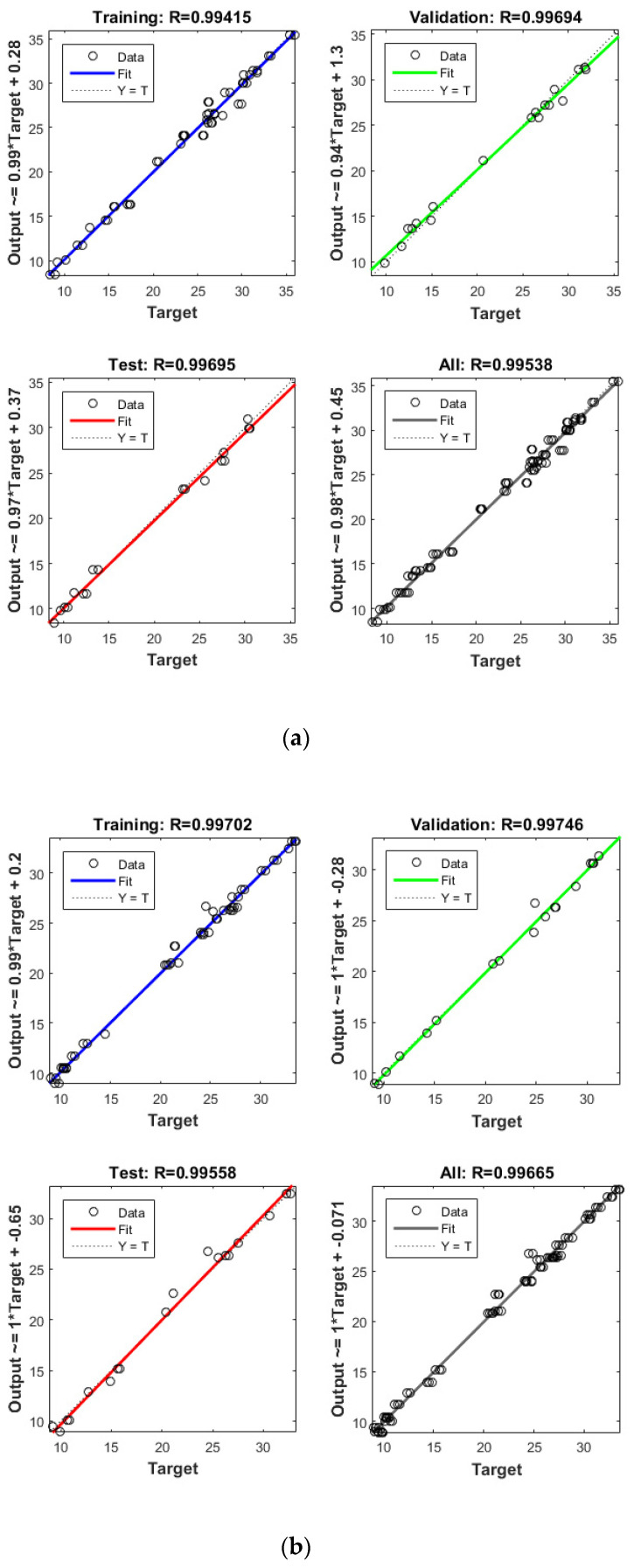

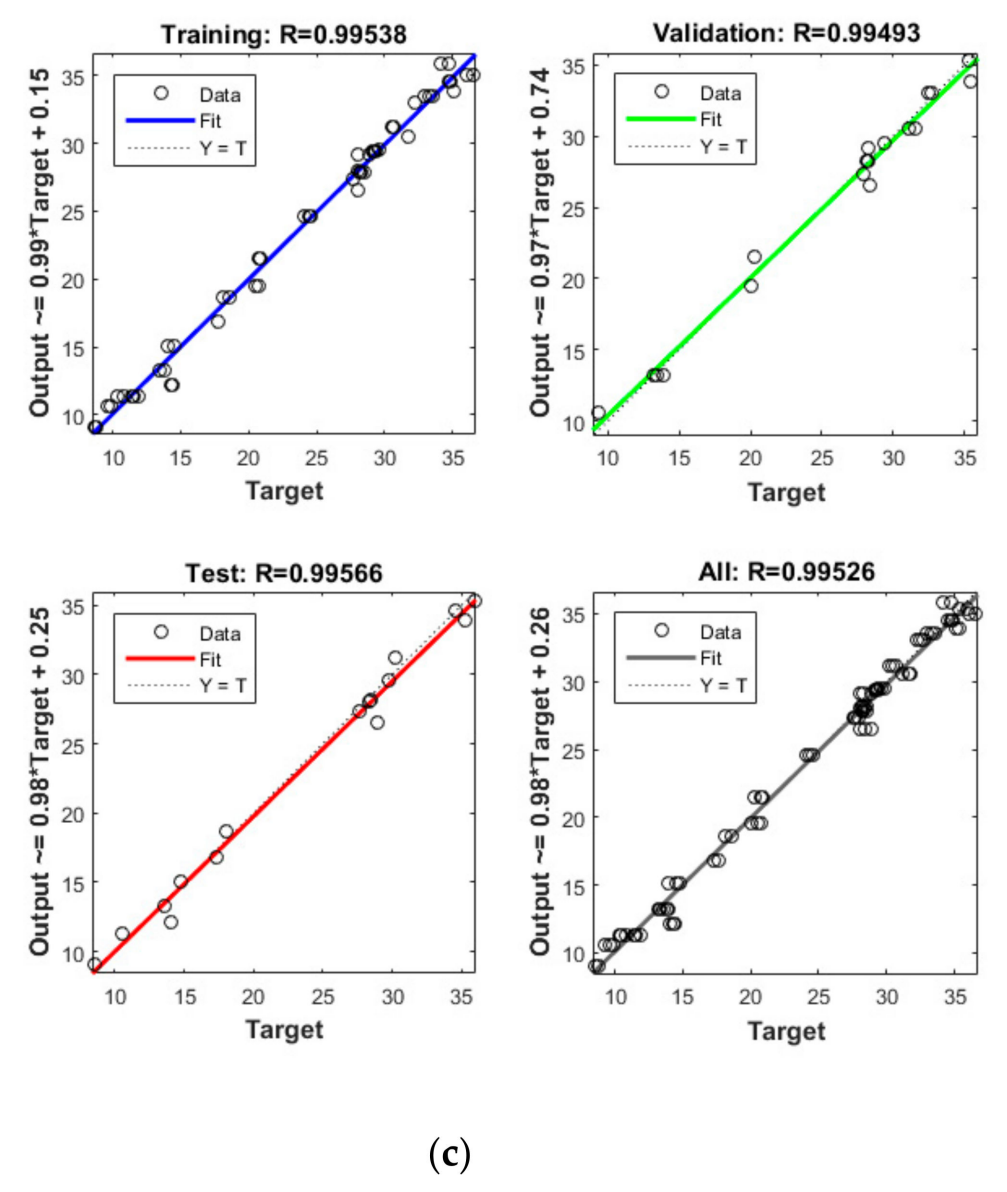
Model prediction versus experimental values for the removal of CBZ (**a**), SMT (**b**) and TRA (**c**) by the first reactor.

**Figure 5:**
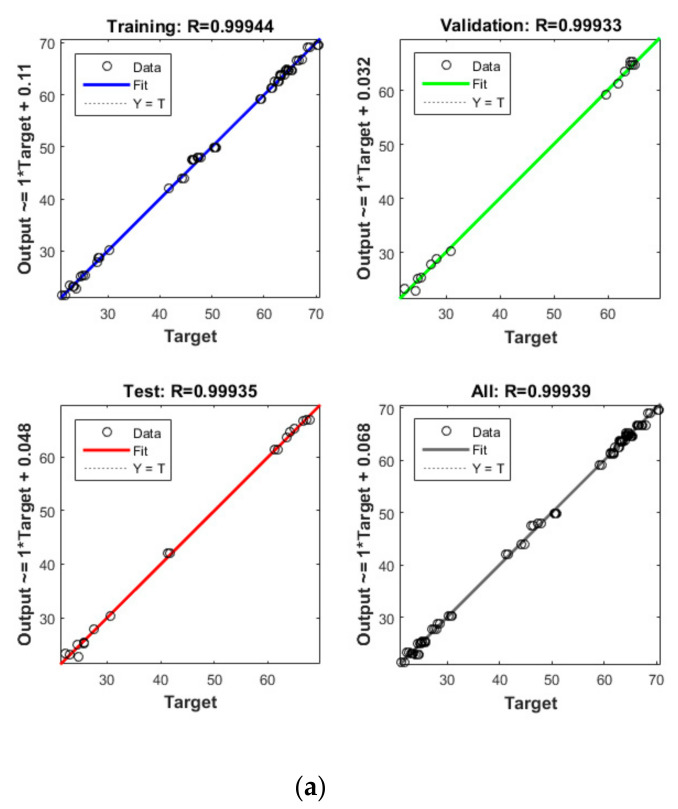

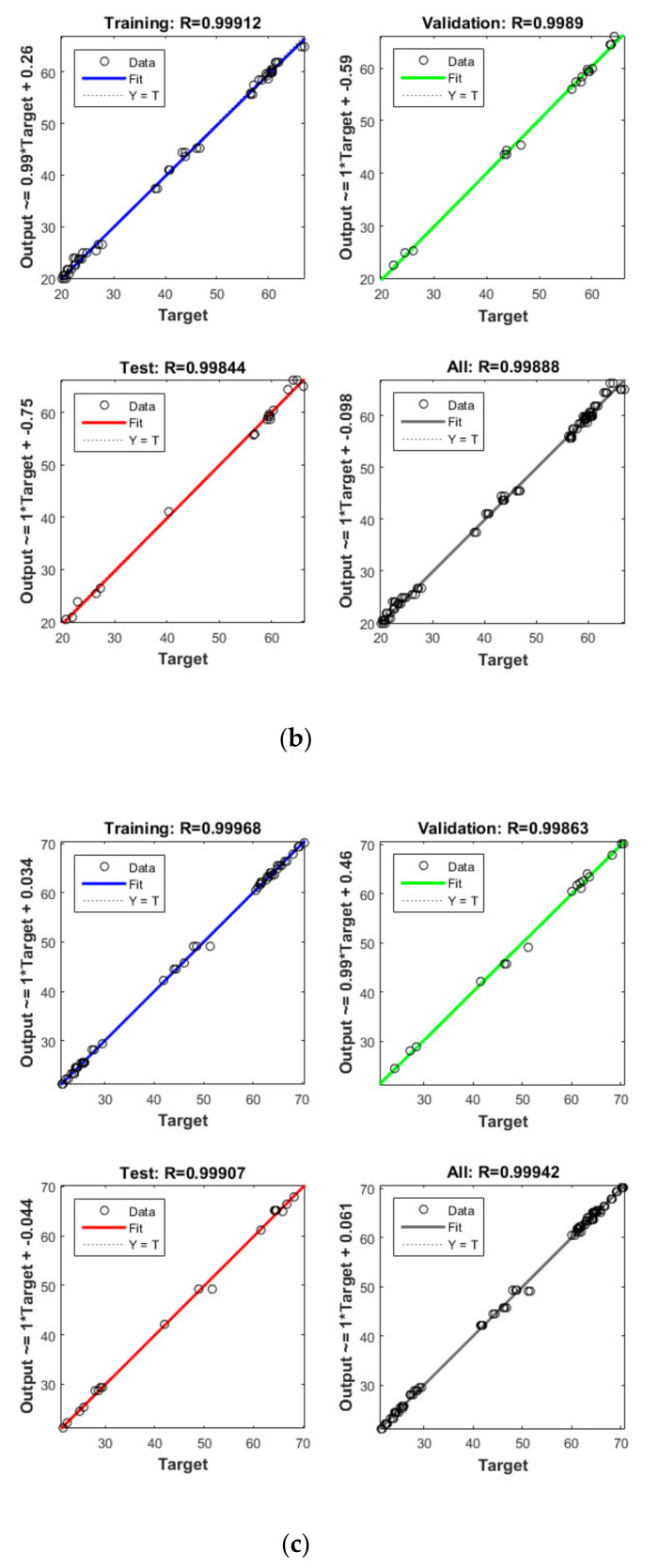
Model prediction versus experimental values for the removal of CBZ (**a**), SMT (**b**) and TRA (**c**) by the second reactor.

This study had two parts. In the first part, CBZ, SMT and TRA were removed with two photobioreactors (one containing *Chaetoceros muelleri* (a marine diatom, first reactor) and another containing *Chaetoceros muelleri* + biochar (second reactor)). Details about PPCP removal with both reactors are shown in Table 2 and Table 3. The performance of each reactor was optimised with the RSM and ANN. Table 4 and Figure 2, Figure 3, Figure 4 and Figure 5 display the details of the optimisation process. In the second part, *Chaetoceros muelleri* was exposed to different concentrations of PPCPs (0–100 mg L^−1^). 

### 3.1. Removal of Pharmaceuticals and Personal Care Products

As shown in Figure 2 and Table 2, the maximum abatement value of CBZ, SMT and TRA were 35.4% (0.070 mg L^−1^), 33.1% (0.066 mg L^−1^) and 36.5% (0.146 mg L^−1^), respectively, in the first reactor, and the initial concentrations of PPCPs (mg L^−1^) and contact times (day) were 0.2 and 8.5 (CBZ), 0.2 and 10.5 (SMT) and 0.4 and 8.5 (TRA). Xiong et al. [38] removed 35% and 28% of CBZ after 10 days of using *Chlamydomonas mexicana* and *Scenedesmus obliquus*, respectively. Approximately 17.3–29.3% of SMT was removed by *Scenedesmus obliquus* in 12 days [39], and 45% of TRA was removed by *Desmodesmus* sp. RUC2 [40]. Moderate-to-low degradation was observed in the removal of CBZ and TRA through an alga-based treatment method [41]. These PPCPs are resistant to photolysis, and thus the removal efficiency of the pollutants are low [42]. Biodegradation and bioaccumulation are the main mechanisms in PPCP removal [43].

As shown in Figure 3 and Table 3, the performance of *Chaetoceros muelleri* in the presence of biochar (second reactor) was higher than that in the first reactor. The maximum removal rates of CBZ, SMT and TRA were 70.2% (0.421 mg L^−1^), 66.4% (0.398 mg L^−1^) and 70.1% (0.420 mg L^−1^) in the second reactor, in which the initial concentration of PPCPs (mg L^−1^) and contact time (day) were 0.6 and 8.5, respectively. These results can be explained by the adsorption of PPCPs by biochar as well as improvement in the microalgal community in the presence of biochar. Ndoun et al. [44] removed 40% of pharmaceutical by biochar at neutral pH. Magee et al. [45] stated that biochar can absorb nutrients on its surface and attracts and immobilises algae on its surface. Liao et al. [46] stated that the microorganism communities can be enhanced in the presence of biochar. Zhu et al. [47] reported that biochar can enhance the biological degradation of pollutants.

RSM was used to optimise the removal performance of the both reactors. In terms of actual results and significant results at *p* < 0.5, the final equations for the removal of CBZ, SMT and TRA by the first reactor were Equations (10)–(12), respectively.

14.08 − 10.97A + 53.77B + 4.97A^2^ − 0.62A^3^ + 0.02A^4^ (10)

10.57 + 3.67A − 5.34B − 0.11A^2^ (11)

5.06 − 4.03A + 94.70B + 3.17A^2^ − 0.42A^3^ + 284.85B^3^ + 0.01A^4^ (12)

where *A* is the contact time and *B* is the initial concentrations of the micropollutants.

In the first reactor, the maximum removal efficiencies of CBZ (33.5%, 0.077 mg L^−1^), SMT (31.0%, 0.071 mg L^−1^) and TRA (36.5%, 0.083 mg L^−1^) were obtained at optimum contact time of 8.5 days and an initial MP concentration of 0.23 mg L^−1^ with RSM.

The final equations for the removal of CBZ, SMT and TRA in the second reactor were Equations (13)–(15), respectively.

39.53 − 16.56A − 42.11B + 7.46A^2^ − 7.75AB^2^ − 0.89A^3^ + 0.63A^2^B^2^ + 0.03A^4^ (13)

31.52 − 14.36A − 6.94B + 6.61A^2^ − 0.77A^3^ + 0.02A^4^ (14)

32.53 − 15.52A + 11.01B + 7.18A^2^ − 0.85A^3^ + 0.03A^4^ (15)

In the second reactor, the maximum elimination efficiencies of CBZ (68.9%, 0.330 mg L^−1^), SMT (64.8%, 0.331 mg L^−1^) and TRA (69.3%, 0.332 mg L^−1^) were obtained at optimum contact time of 8.1 days and initial MP concentration of 0.48 mg L^−1^ through RSM. By comparing optimisation rates of both reactors, we were able to demonstrate that the biochar+marine diatom (second reactor) removed high amounts of PPCPs in a short time and at high initial MP concentrations.

As shown in Table 4, the *R*^2^ (for experiments) and predicted *R*^2^ were higher than 0.9, showing that the performance of both reactors can be optimised by the RSM. Khalid et al. [48] used the RSM in optimising wastewater treatment using *Chlorella sorokiniana* (microalgae). *R*^2^ (for experiments) and predicted *R*^2^ were higher than 0.9, which are in line with those in the current study. The distribution of actual data versus predicted data are shown in Figures S3 and S4.

**Figure 6:**
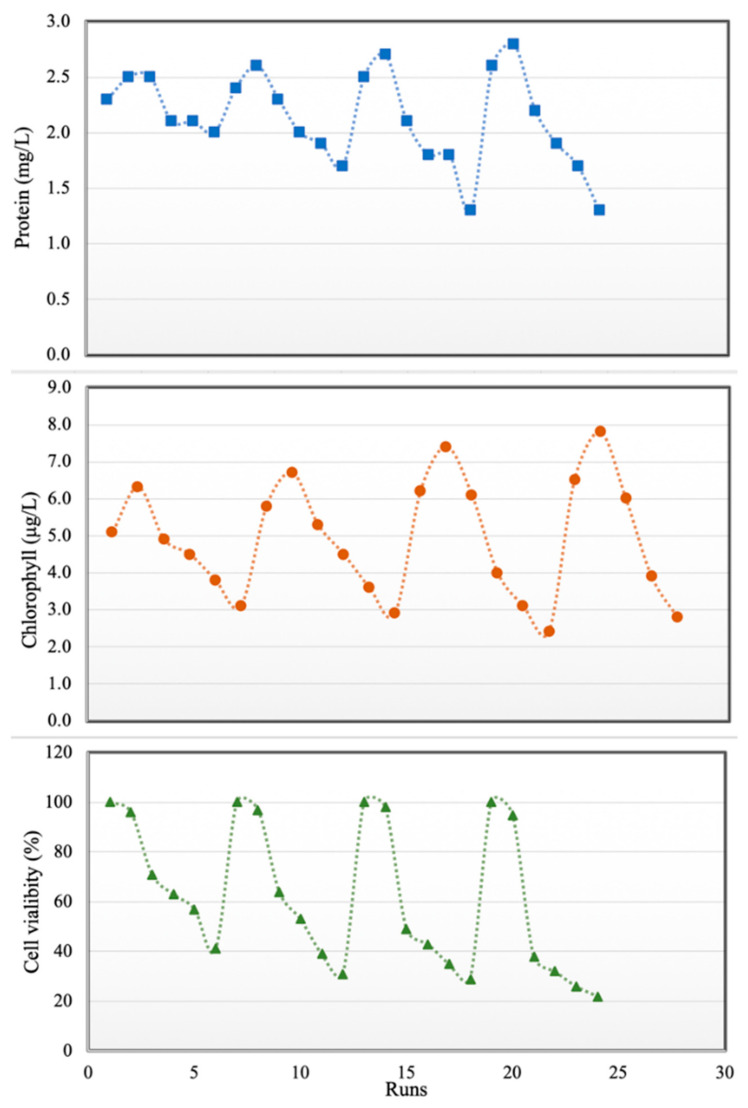
Effects of PPCPs on proteins (**top**), total chlorophyll (**middle**) and cell viability (**bottom**).

Apart from the RSM, an ANN was used in optimising the performance of both reactors. High *R*^2^ (more than 0.99) and reasonable *MSE* (less than 0.80) in the optimisation of all runs (Table 4 and Figure 5 and Figure 6) showed that the ANN could optimise the removal performance of both reactors in a logical way. Figure 5 and Figure 6 display the plots of the experimental data in comparison with the anticipated data and indicate a reasonable distribution of points around the X = Y line in a narrow area. The *MSE* values obtained by using the Levenberg–Marquardt method and selecting different functions and error histograms are shown in Figures S5 and S6 for the firth reactor and Figures S7 and S8 for the second reactor. Training was completed after 22 (CBZ), 19 (SMT) and 21 (TRA) epochs in the first reactor and after 27 (CBZ), 8 (SMT) and 31 (TRA) epochs in the second reactors. These results showed that the ANN model was effectively trained at the end of the training phase [4].

### 3.2. Effects of Pharmaceuticals and Personal Care Products Concentrations on Chaetoceros Muelleri

The combined effects of some pharmaceutical micropollutants on marine diatom have not been reported in previous studies. Therefore, in the second part of this study, water was contaminated with CBZ, SMT and TRA with total PPCP concentrations of 0–100 mg L^−1^. As shown in Figure 6, protein content and total chlorophyll increased with PPCP concentration up to 40 mg/L, and PPCP concentrations of up to 40 mg/L did not have any significant effect on cell viability. Xiong et al. [38] reported that increase in chlorophyll content may enable microalgae to reduce the accumulated reactive oxygen species in chloroplasts in the presence of low amounts of PPCPs. Another reason for increases in protein content and total chlorophyll in diatoms in the presence of low PPCP concentrations is the inductive influence of pharmaceutically active compounds on cells (hormesis) [49]. In the current study, when PPCP concentration exceeded 40 mg/L, cell viability, protein content and total chlorophyll decreased. This result is in line with those of Saygideger and Okkay [25]. Tsiaka et al. [27] stated that algae treated for 24, 48, 72 and 96 h with carbamazepine (more than 10 mg L^−1^) showed an increasing in levels of carotenoids. Zhang et al. [49] expressed that low concentrations of some pharmaceuticals (diclofenac and ciprofloxacin less than 30 mg L^−1^) have a positive impact on chlorophyll *a* accumulation and increasing algae growth. Diclofenac at concentration of more than 40 mg L^−1^ had a 70% inhabitation rate for *Chlorellapyrenoidosa* growth (microalgae). These organic contaminants may cause interference both with the synthesis of protochlorophyll and its subsequent conversion to chlorophyll [26] in high concentrations. Furthermore, long time contact (96 h) with a high amount of PPCP had more negative effects on microalgae during our study. In this study minimum cell viability (22%), chlorophyll (1.5 µg L^−1^) and protein content (1.3 mg L^−1^) of *Chaetoceros muelleri* were recorded at the contact time (96 h) and micropollutant concentration (100 mg L^−1^). Azevedo et al. [50] expressed that the inhibitory impact of ciprofloxacin on the growth of cyanobacteria was accentuated after 48 h. The drug should penetrate the cell for its act, it means taking time to get maximum effects of PPCPs on the cell. Changes in protein, chlorophyll contents and cell viability are shown in Figure 6. The summary of the experiments for determining the impact of PPCPs on microalgae is shown in Table 5.

### 3.3. Adsorption Isotherm Study for the Removal of Pharmaceuticals and Personal Care Products by Biochar

Based on the biochar characteristics analysis, the BET surface area (m^2^ g^−1^), Langmuir surface area (m^2^ g^−1^), micropore area (m^2^ g^−1^) and micropore value (cc/g) were 702, 1171, 301 and 0.19, respectively.

Langmuir isotherm was reached by plotting 1/(x/m) against (1/C_e_) and using Equation (16). Based on the Langmuir isotherm (Table 6), the maximum adsorption capacities (q_m_, mg g^−1^) were 16.6 (CBZ), 13.9 (SMT) and 9.7 (TRA), and the *R*^2^ values were 0.918, 0.904 and 0.902, respectively. Ndoun et al. [44] reported a q_m_ value of 17 mg g^−1^, and Kim et al. [51] reported a value of 0.99 for the removal of PPCPs by biochar, which are nearly equal to the findings of the current study.

(16) $$\frac{x}{m}=\;\frac{abC_{e}}{\left(1+bC_{e}\right)}$$

where *x/m* is the adsorbed the mass of the adsorbate (mg g^−1^), *a* and *b* are empirical constants and *C_e_* denotes the adsorbate concentration after the adsorption process (mg L^−1^).

Freundlich isotherm factors were obtained by plotting log (x/m) against log (C_e_). The liner equation for the calculation parameters of the Freundlich isotherm is provided in Equation (17). Based on the Freundlich isotherm, the maximum adsorption capacities (K_f_, mg g^−1^) were 0.39 (CBZ), 0.31 (SMT) and 0.26 (TRA), and the *R*^2^ values were 0.921, 0.913 and 0.931, respectively. Ndoun et al. [44] reported a K_f_ value of 0.33 mg g^−1^, and Kim et al. [51] reported a value of 0.93 for the removal of PPCPs by biochar, which are consistent with the findings of the current study. Therefore, both isotherms could explain PPCP removal by biochar.

(17) $$\frac{x}{m}=\;K_{f}C_{e}^{1/n}$$

where *x/m* is the adsorbed mass of the adsorbate (mg g^−1^), *K_f_* is the capacity factor, 1/*n* is the intensity parameter and *C_e_* is the adsorbate concentration in equilibrium after the adsorption process (mg L^−1^).

## 4. Conclusions

High concentrations of persistent PPCPs may affect the bioremediation process of marine microalgae; consequently, integrated biochar and *Chaetoceros muelleri* may improve elimination performance. Two photobioreactors were employed, one of which comprised *Chaetoceros muelleri* (first reactor) and another comprised biochar and *Chaetoceros muelleri* (second reactor). The vital findings of the current research are as follows:

1. During running the first photobioreactor, the maximum abatement of CBZ, SMT and TRA was 90.5%, 93.5% and 88.7%, respectively, 35.4% (0.070 mg L^−1^), 33.1% (0.066 mg L^−1^) and 36.5% (0.146 mg L^−1^), respectively, on the contact time 8.5 to 10.50 days and the initial concentration of PPCPs of 0.20 to 0.40 mg L^−1^.
2. The optimum removal of CBZ, SMT and TRA was 70.2% (0.421 mg L^−1^), 66.4% (0.398 mg L^−1^) and 70.1% (0.420 mg L^−1^) during running the second reactor, respectively, on the contact time 8.5 days and the initial concentration of PPCPs of 0.60 mg L^−1^.
3. Based on the optimisation with RSM, the performance of the second reactor was much more than the first reactor. And maximum removal of CBZ (68.9%, 0.330 mg L^−1^), SMT (64.8%, 0.331 mg L^−1^) and TRA (69.3%, 0.332 mg L^−1^) was achieved at optimum contact time (8.1 d), and initial concentrations of MPs (0.48 mg L^−1^).
4. By increasing the PPCPs concentration up to 40 mg L^−1^, protein and chlorophyll of marine diatom were increased. However, the protein, chlorophyll and cell viability were decreased by increasing the PPCP concentration from 40 mg L^−1^ to 100 mg L^−1^.
5. The *R*^2^ values and *MSE* values were >0.99 and <0.90 during optimising removal of PPCPs with both reactors by ANN.
6. Both Freundlich and Langmuir isotherms are proper for clarifying PPCPs adsorption by biochar.

## Figures and Tables

**Table 1:** Characteristics of the pharmaceuticals.

Pharmaceuticals	Structure	CAS Number	Molecular Weight (mg)	References
Carbamazepine (CBZ)	C_15_H_12_N_2_O	298-46-4	235.3	[29]
Sulfamethoxazole (SMT)	C_10_H_11_N_3_O_3_S	723-46-6	253.3	[29]
Tramadol (TRA)	C_16_H_25_NO_2_	27203-92-5	263.37	[9]

**Table 2:** Response values for different independent factors in the first reactor.

Run	Independent Factors		Average Removal of Organic Micropollutants					
	Contact Time (Day)	Initial Concentration (mg/L)	CBZ (%)	CBZ (mg L^−1^)	SMT (%)	SMT (mg L^−1^)	TRA (%)	TRA (mg L^−1^)
1	0.5	0.2	14.9	0.029	12.3	0.024	13.6	0.027
2	0.5	0.4	13.2	0.052	11.6	0.046	14.3	0.057
3	0.5	0.6	11.4	0.068	9.2	0.055	10.5	0.063
4	0.5	0.8	10.1	0.080	9.8	0.078	9.6	0.076
5	0.5	1.0	8.9	0.089	9.8	0.098	8.8	0.088
6	2.5	0.2	17.3	0.034	15.2	0.030	18.1	0.036
7	2.5	0.4	15.2	0.060	14.5	0.058	17.3	0.069
8	2.5	0.6	12.8	0.076	10.3	0.061	14.0	0.084
9	2.5	0.8	12.0	0.096	10.9	0.087	13.4	0.107
10	2.5	1.0	9.2	0.092	10.4	0.104	11.9	0.119
11	4.5	0.2	26.3	0.052	24.5	0.049	28.2	0.056
12	4.5	0.4	29.6	0.118	27.0	0.108	28.4	0.113
13	4.5	0.6	26.5	0.159	24.1	0.144	24.6	0.147
14	4.5	0.8	23.2	0.185	21.8	0.174	20.8	0.166
15	4.5	1.0	20.7	0.207	20.4	0.204	20.5	0.205
16	6.5	0.2	31.9	0.063	27.2	0.054	35.4	0.070
17	6.5	0.4	30.2	0.120	25.3	0.101	32.2	0.128
18	6.5	0.6	28.1	0.168	24.0	0.144	30.5	0.183
19	6.5	0.8	27.8	0.222	21.5	0.172	29.6	0.236
20	6.5	1.0	25.6	0.256	20.6	0.206	28.2	0.282
21	8.5	0.2	35.4	0.070	31.2	0.062	35.9	0.071
22	8.5	0.4	33.3	0.133	30.3	0.121	36.5	0.146
23	8.5	0.6	30.2	0.181	28.1	0.168	33.0	0.198
24	8.5	0.8	26.3	0.210	26.6	0.212	28.1	0.224
25	8.5	1.0	23.3	0.233	25.7	0.257	27.9	0.279
26	10.5	0.2	31.7	0.063	33.1	0.066	34.8	0.069
27	10.5	0.4	30.4	0.121	32.8	0.131	34.8	0.139
28	10.5	0.6	27.5	0.165	30.5	0.183	31.1	0.186
29	10.5	0.8	26.9	0.215	27.2	0.217	29.4	0.235
30	10.5	1.0	26.0	0.260	26.8	0.268	28.1	0.281

**Table 3:** Response values for different independent factors in the second reactor.

Run	Independent Factors		Average Removal of Organic Micropollutants					
	Contact Time (Day)	Initial Concentration (mg/L)	CBZ (%)	CBZ (mg L^−1^)	SMT (%)	SMT (mg L^−1^)	TRA (%)	TRA (mg L^−1^)
1	0.5	0.2	27.9	0.055	24.9	0.049	27.3	0.054
2	0.5	0.4	25.2	0.100	23.3	0.093	25.6	0.102
3	0.5	0.6	24.4	0.146	21.9	0.131	24.0	0.144
4	0.5	0.8	23.1	0.184	21.5	0.172	23.9	0.191
5	0.5	1.0	21.9	0.219	20.1	0.201	21.4	0.214
6	2.5	0.2	30.3	0.060	27.9	0.055	29.5	0.059
7	2.5	0.4	28.2	0.112	26.6	0.106	28.0	0.112
8	2.5	0.6	25.8	0.154	22.9	0.137	25.6	0.153
9	2.5	0.8	24.0	0.192	22.5	0.180	24.5	0.196
10	2.5	1.0	22.2	0.222	20.8	0.208	22.4	0.224
11	4.5	0.2	47.3	0.094	43.9	0.087	48.6	0.097
12	4.5	0.4	50.6	0.202	46.8	0.187	51.3	0.205
13	4.5	0.6	46.5	0.279	43.9	0.263	46.8	0.280
14	4.5	0.8	44.2	0.353	40.3	0.322	44.1	0.352
15	4.5	1.0	41.7	0.417	38.5	0.385	41.8	0.418
16	6.5	0.2	61.9	0.123	56.9	0.113	61.8	0.123
17	6.5	0.4	64.2	0.256	60.8	0.243	64.3	0.257
18	6.5	0.6	64.1	0.384	59.5	0.357	63.0	0.378
19	6.5	0.8	61.8	0.494	57.0	0.456	61.1	0.488
20	6.5	1.0	59.6	0.596	56.9	0.569	60.0	0.600
21	8.5	0.2	67.4	0.134	63.6	0.127	68.2	0.136
22	8.5	0.4	68.3	0.273	64.3	0.257	69.4	0.277
23	8.5	0.6	70.2	0.421	66.4	0.398	70.1	0.420
24	8.5	0.8	66.3	0.530	61.9	0.495	66.9	0.535
25	8.5	1.0	63.3	0.633	59.5	0.595	63.7	0.637
26	10.5	0.2	64.7	0.129	60.8	0.121	65.0	0.130
27	10.5	0.4	64.4	0.257	59.9	0.239	64.6	0.258
28	10.5	0.6	65.5	0.393	60.8	0.364	65.1	0.390
29	10.5	0.8	63.9	0.511	59.8	0.478	62.7	0.501
30	10.5	1.0	62.0	0.620	58.2	0.582	61.9	0.619

**Table 4:** Statistical analysis results for the response parameters in RSM and ANN.

Reactor	Resp.	Optimization with RSM				Optimization with ANN					
		R^2^*	Adj. R^2^	Pred. R^2^	SD	R^2^			MSE		
						**Training	Valid.	Test	Training	Valid.	Test
Reactor-1	CBZ	0.976	0.954	0.915	1.73	0.996	0.996	0.994	0.664	0.535	0.532
	SMT	0.928	0.913	0.897	2.33	0.997	0.997	0.995	0.370	0.333	0.653
	TRA	0.993	0.987	0.981	10.2	0.995	0.994	0.995	0.742	0.710	0.863
Reactor-2	CBZ	0.998	0.996	0.991	1.60	0.999	0.999	0.999	0.330	0.470	0.455
	SMT	0.996	0.993	0.983	1.34	0.999	0.998	0.998	0.622	0.484	0.955
	TRA	0.997	0.995	0.988	1.23	0.999	0.998	0.999	0.215	0.584	0.609

R^2^*: Coefficient of determination; Adj. R^2^: Adjusted R^2^; Pred. R^2^: Prediction R^2^; SD: Standard deviation; and MSE: mean squared errors. **Training, validation and test display the divided data in different steps (training, validation and test) of the ANN process.

**Table 5:** Experiments for examining the effects of PPCPs on microalgae.

Runs	Time (Day)	PPCPs Concentrations (mg L^−1^)	Runs	Time (Day)	PPCPs Concentrations (mg L^−1^)
1	24	0	13	72	0
2	24	20	14	72	20
3	24	40	15	72	40
4	24	60	16	72	60
5	24	80	17	72	80
6	24	100	18	72	100
7	48	0	19	96	0
8	48	20	20	96	20
9	48	40	21	96	40
10	48	60	22	96	60
11	48	80	23	96	80
12	48	100	24	96	100

**Table 6:** Langmuir and Freundlich isotherms study for CBZ, AMT and TRA removal by biochar.

Parameters	Langmuir Isotherm			Freundlich Isotherm		
	Q_m_ (mg/g)	b	R^2^	K_f_ (mg/g(L/mg)^1/n^)	1/n	R^2^
CDZ	16.6	0.10	0.918	0.39	0.69	0.921
SMT	13.9	0.23	0.904	0.31	0.42	0.913
TRA	9.7	0.09	0.902	0.26	0.71	0.931

## Supplementary Materials

The following are available online at https://www.mdpi.com/2076-2607/9/1/4/s1; Figure S1: Cultivation microalgae before treatments, Figure S2: The schematic of ANN model, Figure S3: Data distribution for removal of CBZ (a), SMT (b) and TRA (c) by the first reactor; RSM model, Figure S4: Data distribution for removal of CBZ (a), SMT (b) and TRA (c) by the second reactor; RSM model, Figure S5: The MSE plots during modeling with ANN for removal of CBZ (a), SMT (b) and TRA (c) by the first reactor, Figure S6: The error histogram during modeling with ANN for removal of CBZ (a), SMT (b) and TRA (c) by the first re-actor, Figure S7: The MSE plots during modeling with ANN for removal of CBZ (a), SMT (b) and TRA (c) by the first reactor, and Figure S8: The error histogram during modeling with ANN for removal of CBZ (a), SMT (b) and TRA (c) by the first re-actor.
