# Supplementary material for: Removal of Pharmaceutical Micropollutants with Integrated Biochar and Marine Microalgae
Source: Microorganisms. 2020 Dec 22;9(1):4. doi: 10.3390/microorganisms9010004 (PMC7822045; doi:10.3390/microorganisms9010004)
Supplement: Supplementary file 1 [file microorganisms-09-00004-s001.pdf]

Removal of Pharmaceuticals Micropollutants by Integrated Biochar and Marine Microalgae

Amin Mojiri [1,*,†], Maedeh Baharlooeian [2,†], Reza Andasht Kazeroon [3], Hossein Farraji [4] and Ziyang Lou [5]

[1] Department of Civil and Environmental Engineering, Graduate School of Advance Science and Engineering, Hiroshima University, Higashihiroshima 739-8527, Japan
[2] Department of Marine Biology, Faculty of Marine Science and Oceanography, Khorramshahr University of Marine Science and Technology, Khorramshahr, Iran; bbenicka@yahoo.com
[3] Faculty of Civil Engineering, Xi'an University of Architecture and Technology, Xi'an 710055, China; reza.andasht@gmail.com
[4] School of Physical and Chemical Sciences, University of Canterbury, Christchurch, New Zealand; faraji6211@gmail.com
[5] School of Environmental Science and Engineering, Shanghai Jiao Tong University, Shanghai, China; louworld12@sjtu.edu.cn
* Correspondence: amin.mojiri@gmail.com
† These authors contributed equally to this work.

Figure S1 illustrates the cultivation process of micro algae in our study.

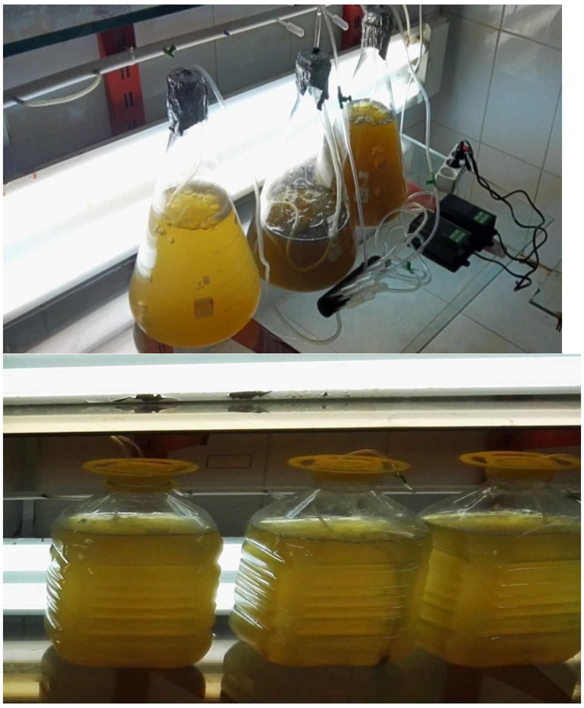

Figure S1. Cultivation microalgae before treatments.

Topology of ANN model in this study is shown in Figure S2.

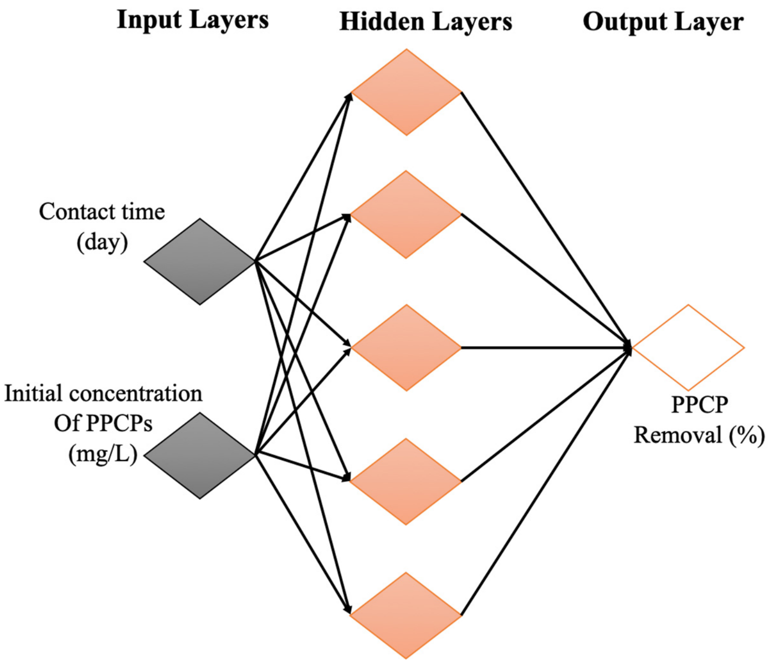

Figure S2. The schematic of ANN model.

The distributions of predicted data and experimental data in RSM model are shown in Figures S3 to S4.

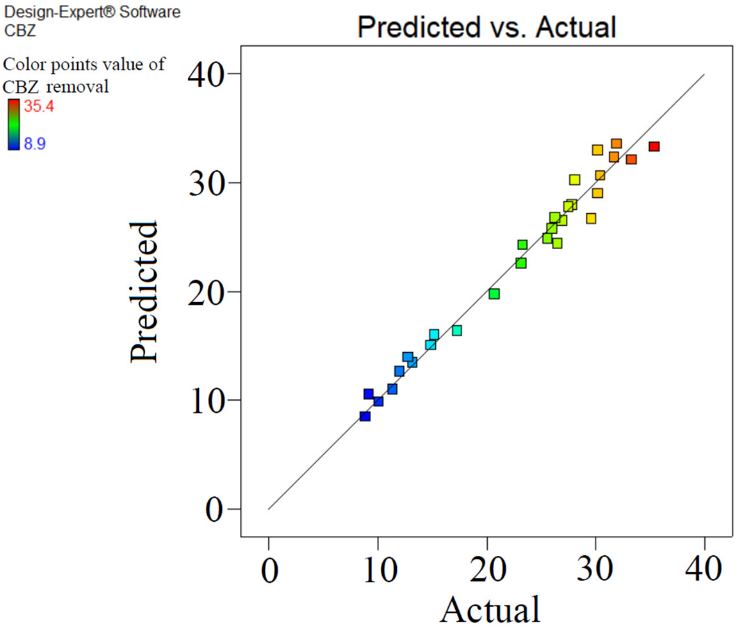
Design-Expert® Software
CBZ
Color points value of
CBZ removal
35.4
8.9
Predicted vs. Actual
Predicted
Actual
0
10
20
30
40

(a)

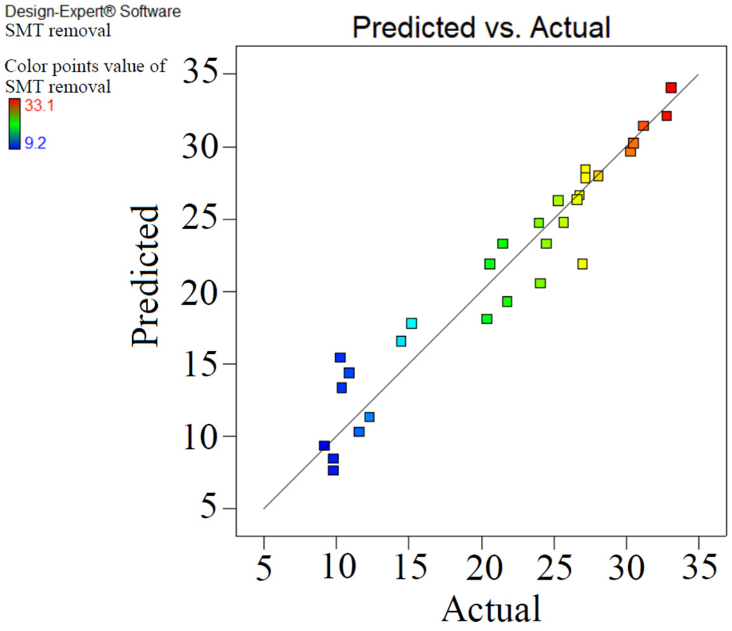
Design-Expert® Software
SMT removal
Color points value of
SMT removal
33.1
9.2
Predicted vs. Actual
Predicted
Actual
5
10
15
20
25
30
35

(b)

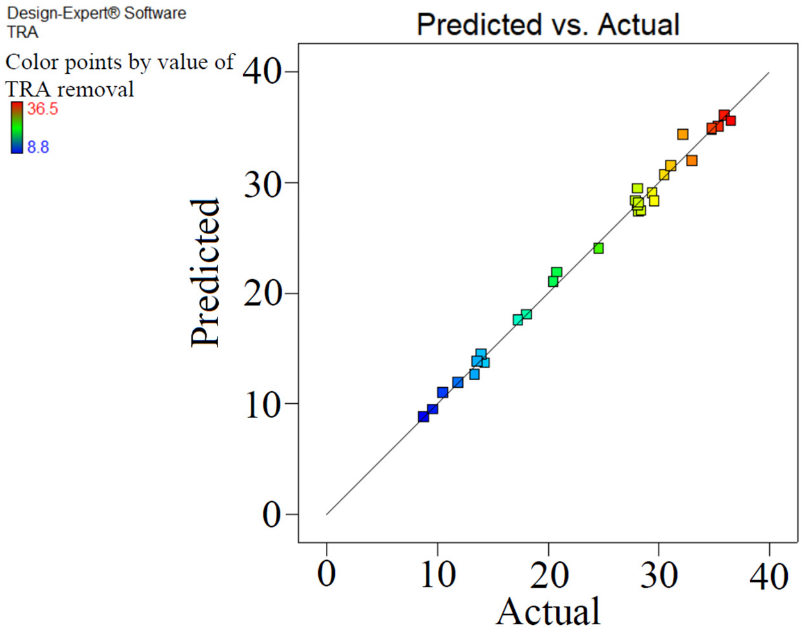

(c)

Figure S3. Data distribution for removal of CBZ (a), SMT (b) and TRA (c) by the first reactor; RSM model.

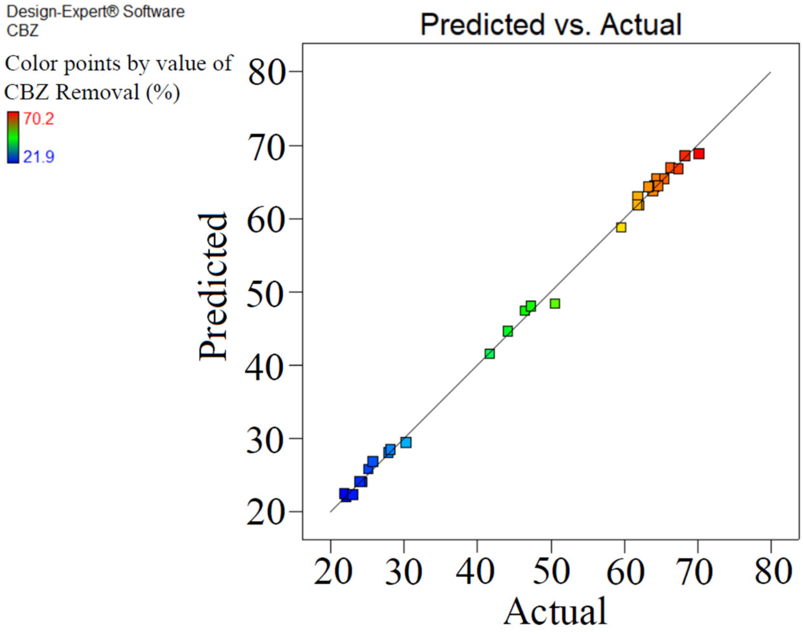

(a)

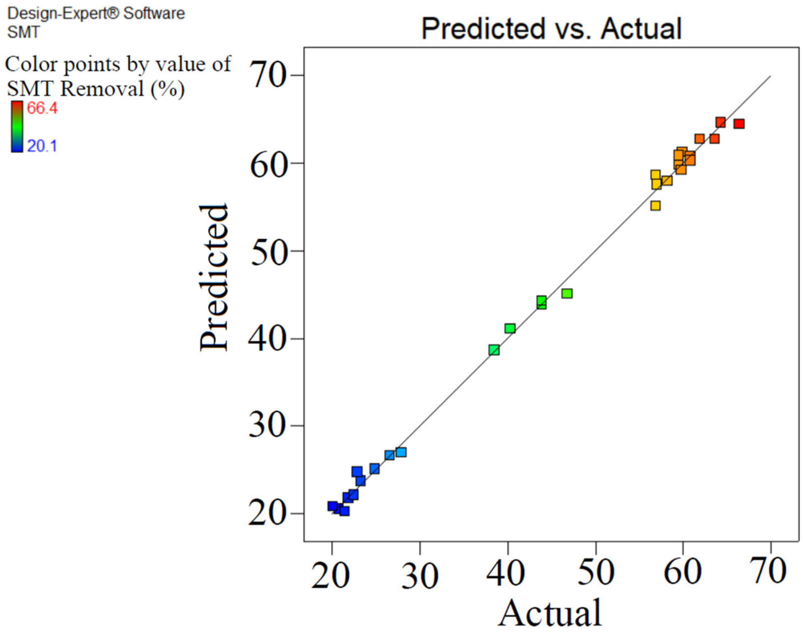

(b)

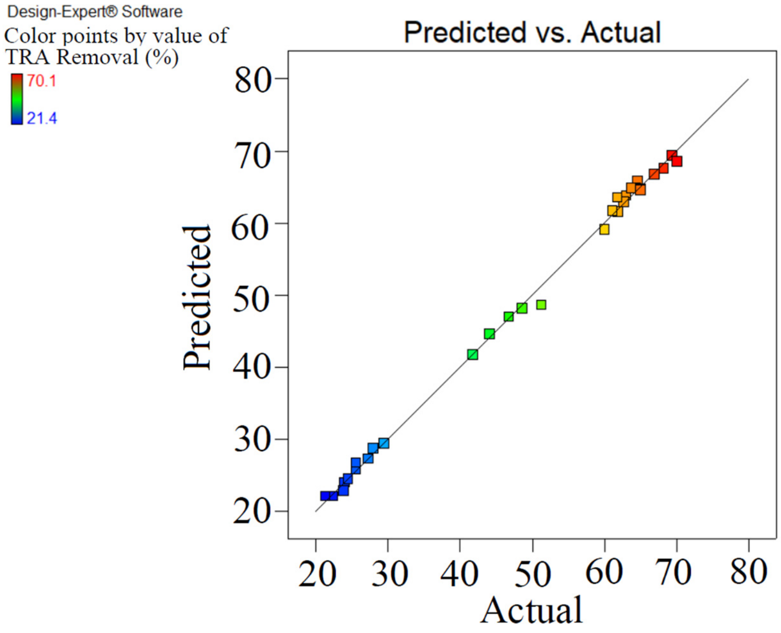

(c)

Figure S4. Data distribution for removal of CBZ (a), SMT (b) and TRA (c) by the second reactor; RSM model.

During ANN modeling for optimising performance of the first reactor, MSE plots and error histogram are shown in Figures A.5 and A.6. And Figures A.7 and A.8 display the error histogram and MSE plots for optimisation performance of second reactor.

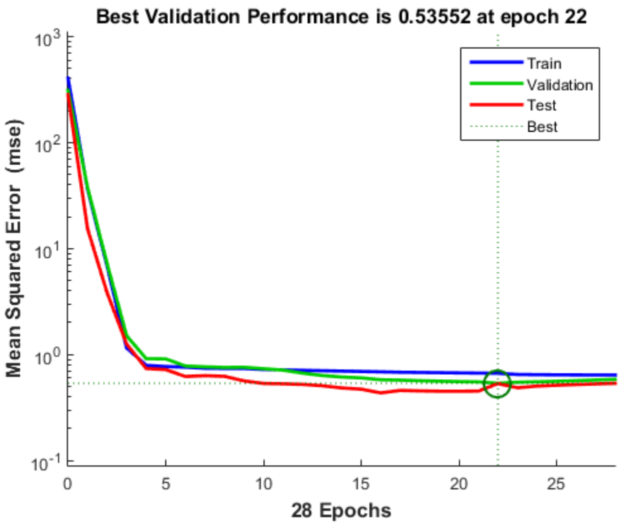

(a)

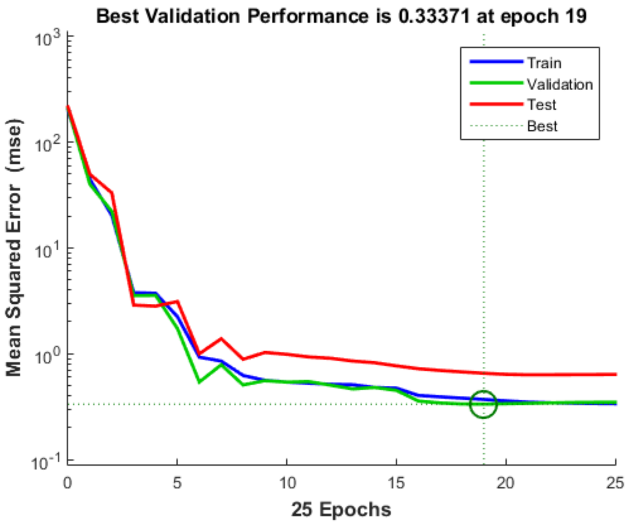

(b)

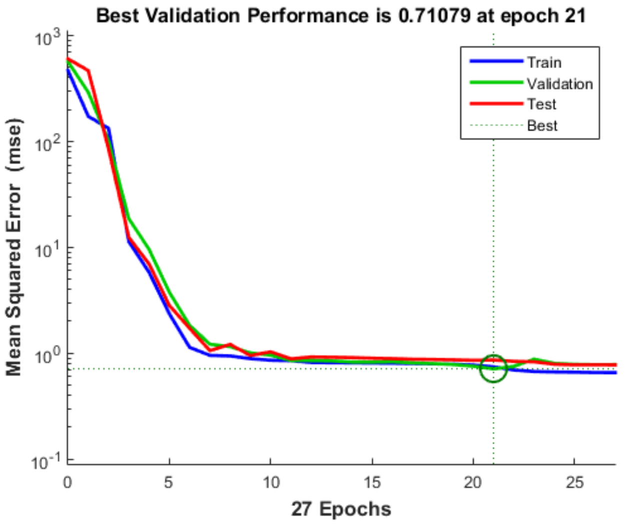

(c)

Figure S5. The MSE plots during modeling with ANN for removal of CBZ (a), SMT (b) and TRA (c) by the first reactor.

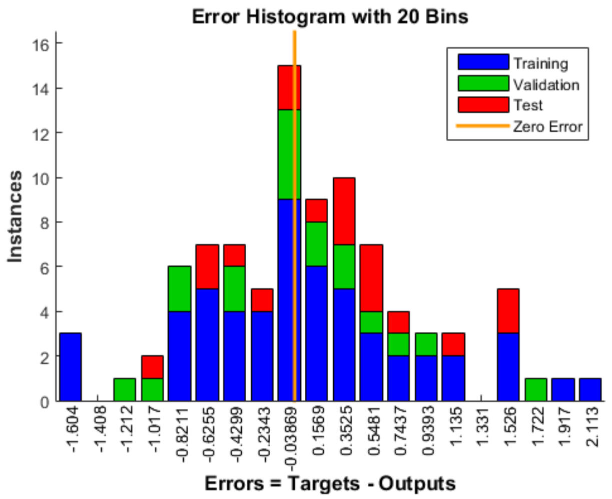

(a)

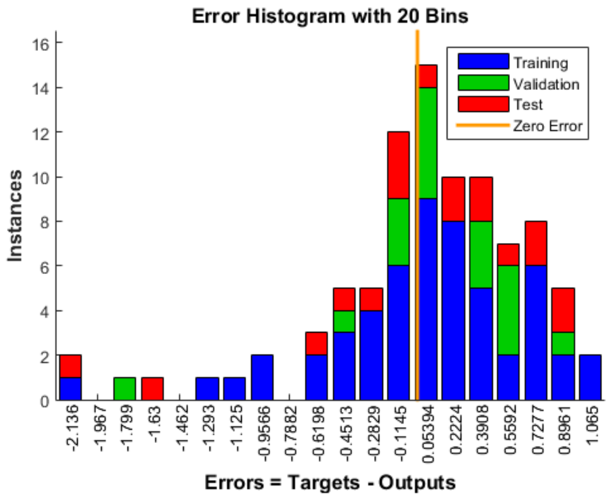

(b)

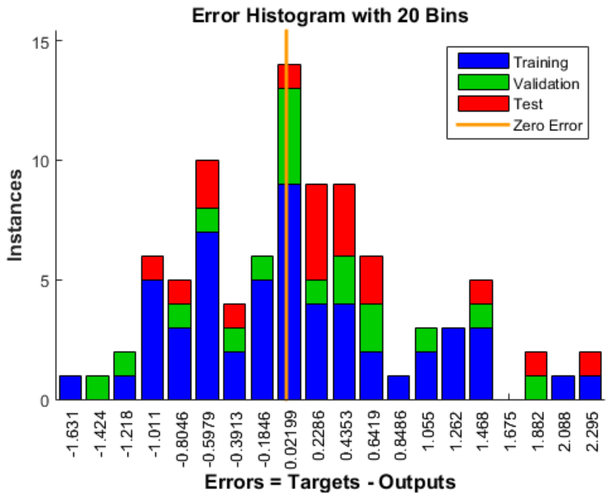

(c)

Figure S6. The error histogram during modeling with ANN for removal of CBZ (a), SMT (b) and TRA (c) by the first reactor.

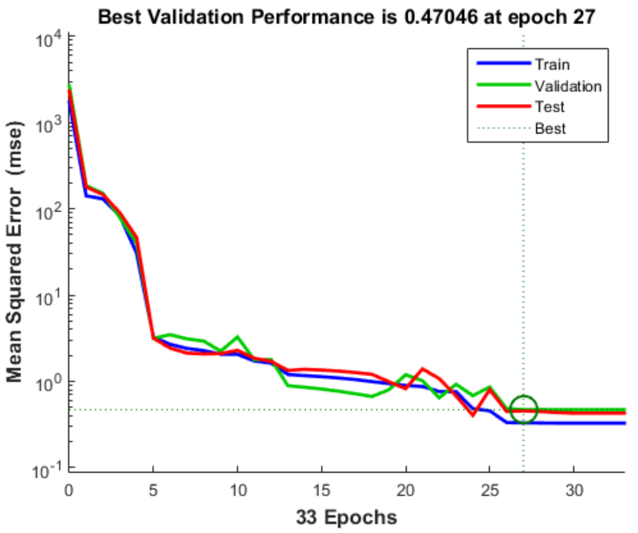

(a)

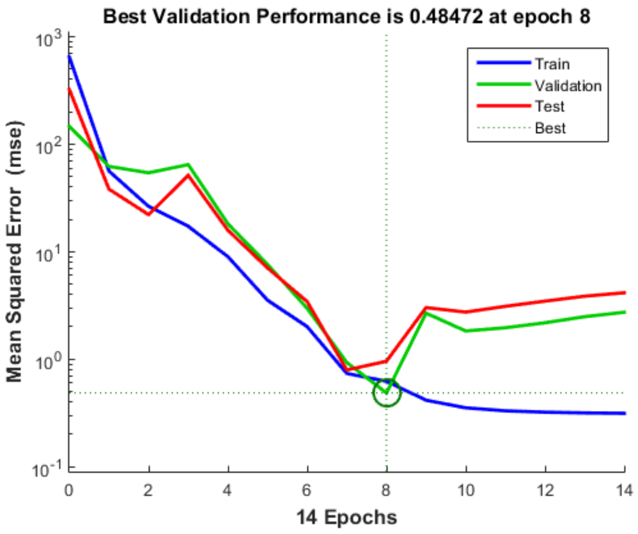

(b)

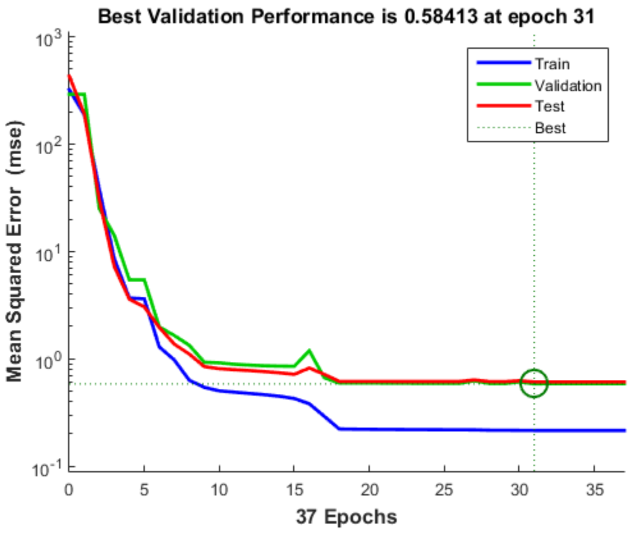

(c)

Figure S7. The MSE plots during modeling with ANN for removal of CBZ (a), SMT (b) and TRA (c) by the first reactor.

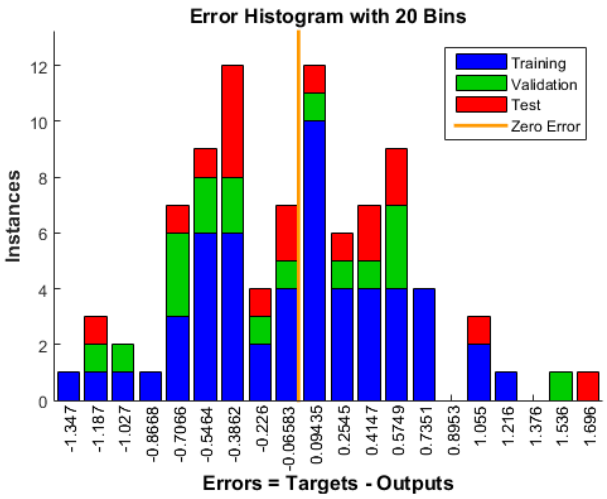

(a)

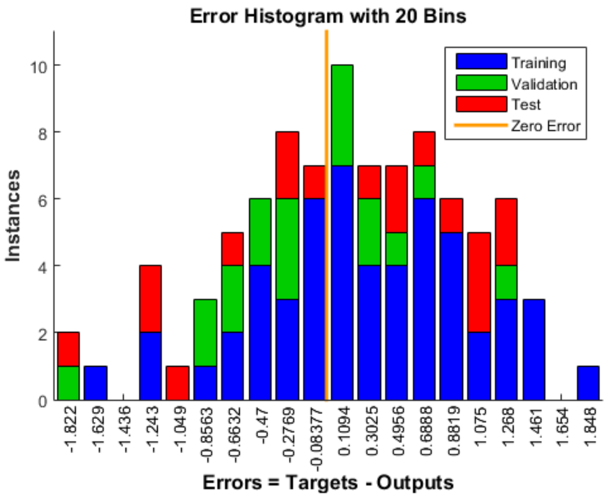

(b)

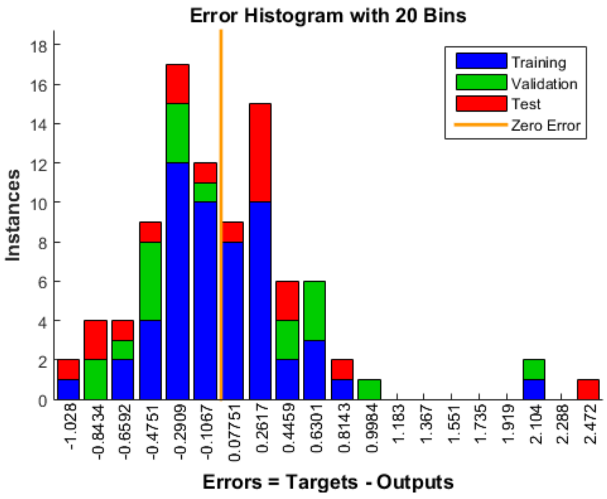

(c)

Figure S8. The error histogram during modeling with ANN for removal of CBZ (a), SMT (b) and TRA (c) by the first reactor.
